# Spontaneous Breathing and Evolving Phenotypes of Lung Damage in Patients with COVID-19: Review of Current Evidence and Forecast of a New Scenario

**DOI:** 10.3390/jcm10050975

**Published:** 2021-03-02

**Authors:** Roberto Tonelli, Alessandro Marchioni, Luca Tabbì, Riccardo Fantini, Stefano Busani, Ivana Castaniere, Dario Andrisani, Filippo Gozzi, Giulia Bruzzi, Linda Manicardi, Jacopo Demurtas, Alessandro Andreani, Gaia Francesca Cappiello, Anna Valeria Samarelli, Enrico Clini

**Affiliations:** 1 University Hospital of Modena, Respiratory Diseases Unit, Department of Medical and Surgical Sciences, University of Modena Reggio Emilia, 41124 Modena, Italy; lucatabbi@gmail.com (L.T.); fantini.riccardo@yahoo.it (R.F.); ivana.castaniere@unimore.it (I.C.); darioandrisani@libero.it (D.A.); fillo.gzz@gmail.com (F.G.); giulibru92@gmail.com (G.B.); linda.manicardi3@gmail.com (L.M.); alessandreani@yahoo.it (A.A.); gaia.cappiello@gmail.com (G.F.C.); annavaleria.samarelli@unimore.it (A.V.S.); enrico.clini@unimore.it (E.C.); 2 Clinical and Experimental Medicine PhD Program, University of Modena Reggio Emilia, 41124 Modena, Italy; eritrox7@gmail.com; 3 Intensive Care Unit, University Hospital of Modena and Reggio Emilia, 41124 Modena, Italy; stefano.busani@unimore.it; 4 Primary Care Department USL Toscana Sud Est-Grosseto, 58100 Grosseto, Italy

**Keywords:** SARS-CoV-2, COVID-19, mechanical ventilation, spontaneous breathing, acute respiratory distress syndrome, acute respiratory failure

## Abstract

The mechanisms of acute respiratory failure other than inflammation and complicating the SARS-CoV-2 infection are still far from being fully understood, thus challenging the management of COVID-19 patients in the critical care setting. In this unforeseen scenario, the role of an individual’s excessive spontaneous breathing may acquire critical importance, being one potential and important driver of lung injury and disease progression. The consequences of this acute lung damage may impair lung structure, forecasting the model of a fragile respiratory system. This perspective article aims to analyze the progression of injured lung phenotypes across the SARS-CoV-2 induced respiratory failure, pointing out the role of spontaneous breathing and also tackling the specific respiratory/ventilatory strategy required by the fragile lung type.

## 1. Introduction

In the early phase of the Severe Acute Respiratory Syndrome Coronavirus 2 (SARS-CoV-2) outbreak, the disproportionate number of patients with severe SARS-CoV-2 disease (COVID-19) and associated acute hypoxic respiratory failure (ARF), compared to the available resources forced clinicians to assist patients with ARF by non-invasive techniques outside intensive care units (ICU), keeping spontaneous breathing preserved despite dramatically impaired gas exchanges [1,2]. Parallelly, when invasive mechanical ventilation (MV) was prompted after non-invasive ventilation failure, a lack of substantial improvement was reported in a significant number of cases [2]. In this scenario, different phenotypes of lung damage have been speculated, starting from the host-driven exaggerated inflammatory response (“cytokine storm”) that may contribute to acute lung injury [2,3]. Furthermore, the intravascular coagulation activation seems to trigger the most severe evolution of COVID-19 and to interfere with the mechanisms of lung repair and wound healing, thus predisposing individuals to aberrant mechanisms of repair and fibrosis [4,5,6]. However, the pathophysiology of COVID-19-induced lung damages may not be limited to the inflammatory and micro-thrombotic hypotheses. Indeed, a recent exploratory study on 39 patients with COVID-19-related ARDS suggested that the hyperinflammatory phenotype is less prevalent, although more severe, in COVID-19 patients than in previous non-COVID-19 cohorts [7]. Thus, in patients with preserved spontaneous breathing, mechanical reasons beyond biochemical causes may be hypothesized in driving lung injury progression. In particular, the role of inspiratory effort in promoting lung damage phenotypes in COVID-19 is may be critical. With this perspective article, we tried to explore the dynamic interaction between spontaneous breathing and lung damage in the COVID-19 model of respiratory failure, forecasting the potential evolution of SARS-CoV-2 induced ARDS, in a new bio-mechanical phenotype of injured lungs.

## 2. COVID-19 and Phenotypes of Lung Damage

Based on physio-pathological hypotheses, patients with COVID-19 pneumonia undergoing mechanical ventilation can be divided into two major phenotypes: “Non-ARDS” type L (low elastance, low ventilation-to-perfusion ratio, low lung weight, low lung recruitability), and “ARDS” type H (high elastance, high right-to-left shunt, high lung weight, high lung recruitability) [1,3]. Type L seems to be the most frequent pattern and exhibits a dissociation between the mechanical characteristics of the respiratory system and the severity of hypoxemia. These patients show a radiological pattern characterized by ground-glass density with subpleural predominance, with only a slight increase in lung weight, normal lung compliance [8], and a loss of hypoxic vasoconstriction resulting in a low ventilation-to-perfusion (VA/Q) ratio [9]. Type H seems to exhibit typical ARDS features, with radiological appearance of bilateral consolidations, decreased compliance of the respiratory system, and increased lung weight [8,9]. Despite these observations, a recent study provides evidence that mechanically ventilated patients with COVID-19 display a form of lung injury similar to classical ARDS, and only 5–7% show static compliance greater than the 95th percentile of those with classical ARDS [9]. In line with these assumptions, the proposed COVID-19-related phenotypes may be considered as the two extremes of a unique evolving disease. Since the evolution of lung damage may eventually include a course leading to prevalent fibrosis [10], these structural and anatomic alterations, if present, result in significant changes of respiratory mechanics with substantial implications for the application of mechanical ventilation and ventilatory setting [11]. Therefore, considering the peculiar mechanical properties of a fibrotic lung, we here propose a further COVID-19-related phenotype, the “fibrotic” type F, showing either fibrotic appearance on CT scan, fragile lung mechanical features and functional derangement resulting from the static strain.

## 3. Molecular and Mechanical Mechanisms Driving COVID-19 Phenotype Transition

Progression from one phenotype to another may depend on the excessive activation of two main pathways: (1) Aggressive inflammatory response to SARS-CoV-2 infection; (2) physical mechanisms driven by the pulmonary stretch.

Virus infection of lung cells may cause a highly inflammatory form of programmed cell death (pyroptosis), with the secretion of cytokines and chemokines [12,13]. In individuals with the dysfunctional immune response, this process may result in a severe local and systemic inflammatory storm [14], activation of coagulation, and several procoagulant pathways (thrombo-inflammation or immune-thrombosis) [15]. So far, COVID-19-related endotheliitis and microcirculatory clot formation were reported in post-mortem studies [16,17,18,19,20]. Progression from type L to type H phenotype can be caused by both further mechanisms of inflammatory amplification overlapping the host inflammatory response phase and by excessive mechanical stress acting on the lung parenchyma sustained by self-inflicted lung injury (SILI) [17,18,19]. At this stage, in patients with phenotype H, the activation of multiple aberrant host pathways might result in impairment of the mechanisms of lung repair, promoting fibrotic changes and driving the progression towards type F [20]. This evolution may be forecasted through a structural change to the lung scaffolding associated with imbalance between profibrotic (TGF-α, TGF-β, interleukin-1β, platelet-derived growth factor) and antifibrotic (prostaglandin E2, keratinocyte growth factor, hepatocyte growth factor) mediators [21,22]. Indirect evidence in animals and experimental models suggest that both vascular lesions with chaotic repair and angiogenic responses [23] and biophysical insult driven by the mechanical ventilation itself or by the excessive activation of respiratory drive [24,25], could have a key role in this evolving process. In vitro and animal model studies show that mechanical stretch of lung epithelial cells results in TGF-α activation and the lung remodeling process after mechanical ventilation [25].

**Figure 1:**
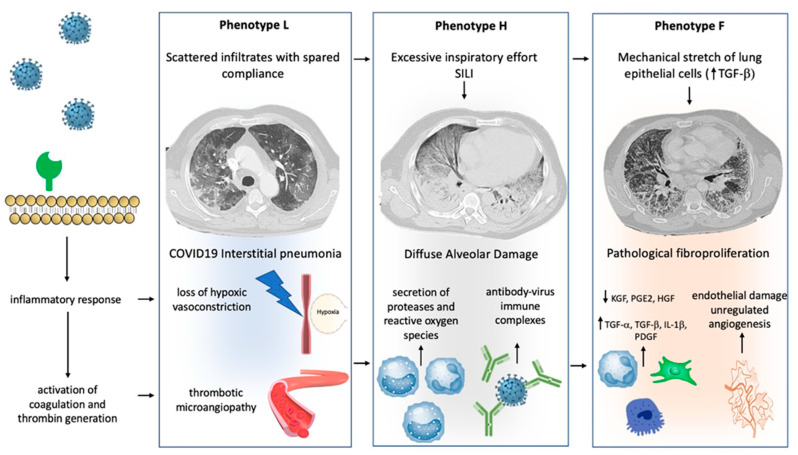
Mechanisms of progression between phenotype: Different hypothetical mechanisms, involving physical stimuli and biological modifications, can determine the progression between COVID-19 phenotypes. Progression from type L to type H may result from excessive inspiratory effort (SILI) and from recruitment of neutrophils into the lung parenchyma with secretion of proteases and reactive oxygen species. Furthermore, a role of activation of Fc receptors immune cells through antibody-virus immune complexes has been hypothesized. Progression from type H to type F results from damage to the scaffolding of the lung and vascular lesions with disorganized repair and imbalance between profibrotic and antifibrotic mediators. Physical factors, such as lung parenchyma stretch, may also contribute via transforming growth factor-beta (TGFB) secretion. See the text for more details.

Figure 1 illustrates the peculiar pathophysiological changes of the lung and the “phenotypes progression” during severe SARS-CoV-2-related respiratory failure.

## 4. The Role of Spontaneous Breathing in COVID-19-Related Lung Injury

In general, maintenance of spontaneous breathing in patients with ARF under ventilatory support has many positive effects such as: Improving oxygenation, preventing the mass loss and atrophy of the peripheral muscles, protecting against the diaphragm dysfunction, reducing the need for pharmacological sedation, and curbing the incidence of delirium [26,27]. Notwithstanding, the critical role of intense respiratory effort and high respiratory drive to foster the progression of lung damage and to favor a myo-diaphragmatic trauma has been demonstrated both in animal models and in humans [28,29,30,31]. In patients with ARDS the inspiratory effort might be affected by different stimuli not always subjected to ventilatory support [32]. In particular different degrees of lung inflammation could influence respiratory drive irrespectively of gas exchange impairment [33]. In animal models of acute lung injury the inflammatory cascade seems to enhance inspiratory effort through the activation of pulmonary C-fibers, vagal-nerve stimulation, and pulmonary stretch receptors inhibition [34]. In patients with COVID-19, the direct invasion of respiratory centers due to SARS-CoV-2, may cause alteration of respiratory drive, thus affecting inspiratory effort [35].

**Figure 2:**
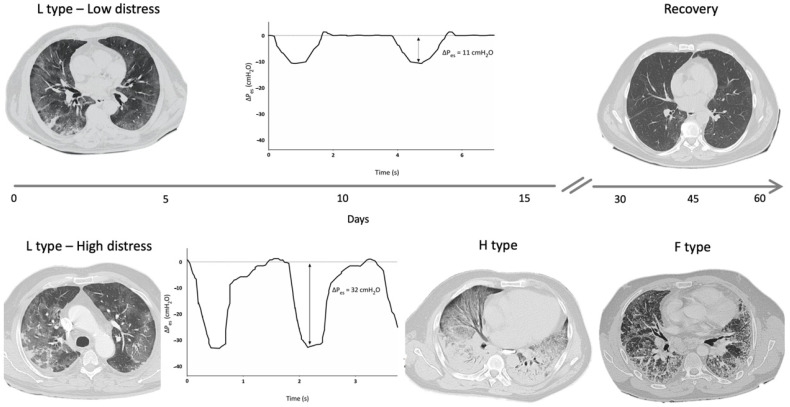
Hypothetical role of inspiratory effort in progression from type L to type H and F: Figure 2 shows DPes monitoring in two patients with SARS-CoV-2 pneumonia undergoing non-invasive ventilation (NIV) with Helmet. Patient 1, at the top of the figure, shows type L phenotype on computed tomography (CT) scan, and modest inspiratory effort (DPes 11 cm H_2_O). Over time, a progressive clinical and radiological improvement with ground glass changes resolutions was recorded. Patient 2, at the bottom of the figure, shows significant inspiratory effort (DPes 32 cm H_2_O). Over time, there was a progression from type L to type H required intubation and invasive mechanical ventilation. Progression from type H to type F was documented after 45 days from Intensive Care Unit admission. DPes: Change in esophageal pressure.

A reliable method to assess changes in pleural space (Ppl) during spontaneous breathing is by using an esophageal balloon catheter to measure esophageal pressure (Pes) as a surrogate of Ppl [36]. A recent study estimated the intensity of spontaneous breathing effort by Pes measurement in 30 patients with hypoxemic ARF [29], and confirmed that vigorous effort is present as in typical ARDS patients [2]. In injured lungs, lung tissue becomes inhomogeneous as a consequence of inflammation and edema, and the distribution of the forces applied to the parenchyma during spontaneous breathing becomes asymmetrical (“solid-like” behavior, opposed to the “liquid-like” behavior typical of healthy lungs) [37,38,39]. In particular, the negative swing in pleural pressure generated by diaphragmatic contraction is not evenly transmitted and tends to concentrate in dependent regions near the diaphragmatic interface, where high local values of PL are developed. This impaired distribution of physical forces during spontaneous breathing causes a pendelluft phenomenon and can result in a local overstretch of the dependent lung [20,40]. In type F lung phenotype, lungs are a patchwork of different tissue elasticities, due to the contiguity of preserved lung tissue and areas of dense anelastic parenchyma [11]. During spontaneous breathing, Ppl swing distribution is even more inhomogeneous and unpredictable, lung tissue deformation occurs unevenly, and some lung areas can reach a harmful stress/strain level. These processes described in COVID-19 injured lungs can result in SILI with diffuse alveolar damage and could be therefore considered as main determinants for the lung phenotype progression over the course and the spectrum of disease severity (Figure 2). In this scenario, it should be noted that a relevant feature in patients experiencing COVID-19 pneumonia is the lack of perceived dyspnea despite severe hypoxemia (the so-called silent hypoxia) [41,42,43]. Recently, a study evaluated airway occlusion pressure (P01), a surrogate measure of respiratory drive, in mechanically ventilated COVID-19 patients. In this cohort of patients, P01 was frequently above 4 cm H_2_O, suggesting high neuronal respiratory drive, high respiratory effort, and excessive respiratory muscles load [44]. It has been described that COVID-19 patients can maintain a (pseudo)normal respiratory rate despite an increase in inspiratory effort, thus indicating that PL and inspiratory effort cannot be estimated by the individual’s breathing frequency [45,46,47,48,49]. Pes monitoring could help in the identification of patients with excessive inspiratory effort who are at risk of SILI and progression towards more serious lung phenotypes.

## 5. How to Assist Spontaneous Breathing across COVID-19-Related Pneumonia Paradigm

Some evidence shows that respiratory assistance, in patients who maintain spontaneous breathing, can modify the magnitude of inspiratory effort and Ppl swings [49,50]. Indeed, non-invasive pressure support ventilation (PSV) reduces inspiratory effort by unloading the respiratory muscles, while continuous positive airway pressure (CPAP) has minimal effect on the pressure generated by inspiration [46]. Recent data suggest that ARDS patients treated with high positive end-expiratory pressure (PEEP) levels, can achieve safe spontaneous breathing under light sedation [51]. In experimental models of ARDS, the recruitment of injured lung by high PEEP acts by converting “solid-like” lung into “fluid-like” lung, allowing a more homogeneous distribution of Ppl swing over the lung surface during tidal volume (Vt) generation, and by lowering the intensity of spontaneous respiratory effort [52,53]. The reduction of inspiratory effort, with high PEEP applications, can partly originate from the increase in lung volume at the end of inspiration, which results in the reduction of diaphragmatic curvature and force-length relationship changes [54]. Indeed, the contractile capacity of the diaphragm is closely related to the length-tension relationship of the muscle. The active tension developed by the muscle during contraction is a function of the length of the muscle at rest before stimulation, which in turn is influenced by lung volume [55]. Ppl swing (i.e., DPes) following phrenic nerve stimulation decreases progressively, with increasing expiratory lung volume, by applying high levels of PEEP [52,56,57]. Moreover, high PEEP application can also reduce inspiratory effort by increased activation of mechanoreceptors (SARs), probably through stabilized lung recruitment [58].

Although the application of PEEP can mitigate the dangerous effects of inspiratory effort, we must even consider that the 3 described lung phenotypes in COVID-19 patients show different physiological pattern responses to the application of pressure in the airways.

In the L type, a “fluid-like” behavior is prevalent. Therefore, since the distribution of the pleural swing is substantially homogeneous along the entire surface of the lung, this lung phenotype could progress towards the H type especially if intense respiratory efforts are present, resulting in negative alveolar pressure, increased lung perfusion, and transmural vascular pressure, and worsening alveolar edema. Thus, even in L type patients, if vigorous inspiratory effort is present under assisted breathing, high PEEP level may be recommended to obtain Ppl swing reduction. In the H type, similar to ARDS, a classic protective ventilation strategy is recommended (tidal volume <6 mL/kg predicted body weight, high PEEP strategy). In the lungs of COVID-19 patients with H type under assisted breathing, a “solid-like” injurious behavior may occur, therefore a higher PEEP strategy (from 10 up to 15 cm H_2_O), converting “solid-like” into “fluid-like” behavior, could be welcomed and protective.

Ventilatory management of the F type is still a challenge for intensivists. Fibrotic lungs have peculiar structural and anatomic features that result in major alterations of the mechanics of breathing [11]. First of all, during lung inflation, fibrotic lungs exhibit anisotropic behavior, because lung tissue does not show the same mechanical properties in all the directions when a physical force (i.e., transpulmonary pressure) is applied. Second, the effect of PEEP can determine the protrusion of the most distensible lung area through the inelastic fibrotic tissue, favoring the formation of “squishy ball” lung areas and the consequent exposure to the organ damage.^11^ Indeed, in patients with fibrotic lung and ARF, retrospective data have shown an association between the use of higher PEEP levels and mortality [59,60]. Third, Vte monitoring is not useful in establishing the risk of volutrauma. Indeed, in fibrotic lungs, even when the global strain during spontaneous breathing is limited, the micro-strain resulting from the “squishy ball” behavior can be harmful. Therefore, a low PEEP level (4–6 cm H_2_O) is recommended in F type lung (lung resting strategy) during spontaneous breathing assistance and measuring of Pes can be useful to monitor the magnitude of effort.

Non-invasive ventilation (NIV), CPAP, and high flow nasal cannula (HFNC) are the most commonly used tools to assist spontaneously breathing patients with ARF due to COVID-19 pneumonia. In addition, the awake prone position has been also considered to play a possible role in the modulation of the inspiratory effort of these patients [61]. Guidelines have suggested so far using NIV in de novo respiratory failure only when managed by an experienced clinical team in the appropriate setting and made no recommendation for or against its use during pandemics [62]. Much of the data guiding the use of NIV in a pandemic context derive from non-randomized trials conducted during SARS-CoV-1 Syndrome (SARS) and Middle Eastern Respiratory Syndrome (MERS) outbreaks. In both scenario, using NIV has been associated with only a slightly reduced risk of subsequent endotracheal intubation (ETI), but without affecting 90-day mortality [63], or even with successful treatment avoiding intubation [64]. Observational trials during the COVID-19 epidemic indicated that using PSV may stabilize the clinical course in patients with mild to moderate ARF [65,66]. The largest retrospective study on the use of non-invasive respiratory support outside ICU in 670 patients with moderate to severe COVID-19-induced ARF (average P/F ratio 138 mmHg) reported a 30% mortality rate when NIV was applied [67]. In a large prospective study on 359 patients admitted to ICU for severe ARF related to SARS-CoV-2 infection, HFNC significantly reduced the intubation rate compared to standard oxygen without affecting mortality [68]. The evidence seems therefore to suggest that NIV and/or HFNC might be helpful in a subset of patients with mild to moderate activation of respiratory drive [65]. It could be recommended, however, to draw attention for close monitoring of the patient’s respiratory effort to tailor pressure support, and to avoid harmful spontaneous breathing. Differently from animal models of ARDS where dynamic strain has proved to be the major mechanism of lung damage, static strain [69], in type F, static strain may assume the greatest importance in causing lung injury [11]. At least theoretically, these assumptions may be also applied to non-invasive ventilatory support, suggesting that when radiological changes following the acute phase of the disease indicate a fibroproliferative evolution, lower PEEP levels may be safer when noninvasive ventilatory management is required. In type F patients, without excessive inspiratory effort, HFNC may be considered in order to increase ventilation efficiency, decrease respiratory rate, and reduce work of breathing [70].

Current trends in the usage, and associated mortality rate, of tools to assist spontaneously breathing patients with COVID-19, are shown in Table 1 [6,42,67,68,71,72,73,74,75,76,77,78].

The prone position aims to reduce V/Q mismatching and shunt fraction in mechanically ventilated patients with ARDS, thus improving hypoxemia, and it is going to be recommended for 12–16 h period long in ventilated COVID-19 patients with moderate to severe ARDS [79,80,81]. Although the available evidence is weak, the physiological benefits of the prone position during mechanical ventilation should also be hypothesized in patients breathing spontaneously. In particular, in patients with “solid-like” lungs, the prone position might result in a more homogeneous Ppl distribution and less harmful lung stretch during spontaneous breathing. Only small studies showed that the prone position improved oxygenation and reduced the need for endotracheal intubation in this population of COVID-19 patients [82]. The largest prospective study on 56 patients with SARS-CoV-2 infection and related pneumonia treated with supplemental oxygen or NIV, showed that an early 3h trial of awake prone position was feasible and effective in improving the oxygenation (P/F) ratio [83]. Interestingly, the increase in blood oxygenation was maintained after resupination in half of the patients. Although the prone position offers a physiological rationale in those patients with L or H lung phenotypes, it seems not to be useful for those patients with fibrotic evolution (F type). Indeed, despite the lack of specific studies exploring this issue, a single trial in patients with lung fibrosis under mechanical ventilation did not show any benefit in improving hypoxia when shifting position from supine to prone [84]. Table 2 summarizes clinical features, mechanical characteristics, and radiological signs of potential phenotype transition across difference clinical scenarios, aside from practical management suggestions.

## 6. Discussion

The mechanisms of severe respiratory distress following the SARS-CoV-2 infection are heterogeneous and still far from being fully understood. The attempt to identify different COVID-19-related lung phenotypes stems from the need to tailor ventilatory strategy on the basis of different physiological features behind. Notwithstanding, disease progression is often unpredictable, and overlap between these phenotypes may occur.

The interaction between inflammatory and mechanical triggers challenges the hypothesis of a dominant mechanism for the disease (i.e., cytokine storm). Among the possible mechanisms of lung phenotype transition, exaggerated inspiratory effort deserves careful attention. Experimental models suggest that SILI could exacerbate damage through several physiological mechanisms (pendelluft phenomenon, negative alveolar pressure edema, unphysiological transpulmonary pressure distribution), thus suggesting that monitoring of inspiratory effort could be crucial in the early recognition of patients at risk for lung damage.

The application of noninvasive respiratory support (namely NIV and HFNC) in spontaneously breathing patients could modify the magnitude of respiratory effort, thus mitigating the progression across the COVID-19 phenotypes. Phenotype transition may proceed up to a fragile, fibrotic, and functionally deranged fibrotic (F) phenotype whose mechanical features require a strategic respiratory/ventilatory approach [11].

## Figures and Tables

**Table 1:** Use of non-invasive respiratory support and related mortality among patients with COVID-19.

Authors	Patients *n*	NIV *n* (%)	Mortality (%)	HFNC *n* (%)	Mortality (%)	NIS *n* (%)	Mortality (%)
Demoule [68]	379	---	---	146 (38.5)	21	146 (38.5)	21
Arentz [71]	21	4 (19)	---	1 (4.7)	---	5 (23.8)	NA
Huang [72]	41	---	---	---	---	10 (24.4)	NA
Wang [65]	138	15 (10.8)	--	---	---	15 (10.8)	NA
Yang [66]	62	29 (56)	79	33 (63)	49	62 (100)	64
Wang [73]	344	34 (10)	79	12 (3.4)	58	46 (13.4)	74
Chen [74]	274	102 (37.2)	75	85 (24.7)	91	187 (68.2)	82
Zhou [75]	191	26 (13.6)	92	41 (21.4)	81	67 (35.1)	85
Guan [42]	1099	---	---	---	---	56 (5)	NA
Wu [76]	201	61 (30.3)	62	---	---	61 (30.3)	62
Bhatraju [77]	24	---	---	10 (41.6)	---	10 (41.6)	NA
Grasselli [6]	1591	137 (8.6)	---	---	---	137 (8.6)	NA
Aliberti [78]	157	---	---	---	---	157 (100)	NA
Cosimo [67]	670	177 (26.4)	31	163 (24.3)	16	340 (50.7)	33
Total	5182	585		491		1143	
Weighted mortality			47.2		26.4		46.1

HFNC, high-flow nasal cannula; NIS, all non-invasive respiratory support including CPAP, continuous positive airways pressure; NIV, non-invasive ventilation; NA not applicable.

**Table 2:** Clinical features, mechanical characteristics, and radiological signs of potential phenotype transition across difference clinical scenarios and practical management suggestions.

Clinical Scenario	Target	Signs of Alert of Phenotype Transition	Practical Suggestions	
			Mandatory	Optional
Spontaneous breathing	Prevent phenotype L to phenotype H transition	High respiratory rate and/or respiratory distress High systemic inflammation Long symptoms onset	Inspiratory effort assessment Assist breathing (consider HFNC trial)	Chest X-ray Consider prone position
Assisted spontaneous breathing (HFNC)	Prevent phenotype L to phenotype H transition	High respiratory rate and/or respiratory distress High systemic inflammation	Chest X-ray Inspiratory effort assessment Decrease inspiratory effort (consider NIV trial)	Chest CT scan Advanced inspiratory effort assessment (P_es_ pressure monitoring) Consider prone position
Assisted spontaneous breathing (NIV)	Prevent phenotype L to phenotype H and phenotype H to phenotype F transition	High respiratory rate and/or respiratory distress Excessively high Vte DissynchronyRadiological appearance of scattered consolidations and/or lung volume reduction	Chest X-ray Advanced inspiratory effort assessment (P_es_ monitoring)Decrease inspiratory effort (trial of pressure support increase) Consider IMV	Chest CT scan Start/optimize sedation Consider prone position
Invasive mechanical ventilation	Prevent phenotype H to phenotype F transition	Worsening of respiratory system compliance (<50 mL/cm H_2_O) Low PEEP response Plateau pressure > 30 cm H_2_O Need for elevated FiO_2_ Low response to pronation Weaning difficulty High systemic inflammation Radiological appearance of fine reticulation/traction bronchiectasis and/or lung volume reduction	Chest CT scan Advanced respiratory mechanics assessment (P_L_ assessment) Ultra-protective ventilatory strategy	Consider ECMO

NIV–Non-invasive mechanical ventilation; HFCN–High flow nasal cannula; CT–Computed tomography; Transesophageal pressure–P_es_; Transpulmonary pressure; ECMO–Extra Corporeal Membrane Oxygenation; PEEP–Positive end-expiratory pressure; Vte–Expiratory tidal volume; IMV–Invasive Mechanical Ventilation.

## Data Availability

Data are available upon request at the Respiratory Intensive Care Unit of the University Hospital of Modena

## References

[1] Marini J.J., Gattinoni L. (2020). Management of COVID-19 Respiratory Distress. JAMA.

[2] Lari F., Giostra F., Guerrini S. (2020). Use of non-invasive ventilation in acute respiratory failure due to SARS-CoV-2 pneumonia: Typing of patients and choice of respiratory support, the role of internal medicine. Ital. J. Med..

[3] Gattinoni L., Chiumello D., Caironi P., Busana M., Romitti F., Brazzi L., Camporota L. (2020). COVID-19 pneumonia: Different respiratory treatments for different phenotypes?. Intensive Care Med..

[4] Tang X., Du R.H., Wang R., Cao T.Z., Guan L.L., Yang C.Q., Zhu Q., Hu M., Li X.-Y., Li Y. (2020). Comparison of Hospitalized Patients with ARDS Caused by COVID-19 and H1N1. Chest.

[5] Wang J., Wang B.J., Yang J.C., Wang M.Y., Chen C., Luo G.X., He W.F. (2020). Research advances in the mechanism of pulmonary fibrosis induced by coronavirus disease 2019 and the corresponding therapeutic measures. Zhonghua Shao Shang Za Zhi.

[6] Grasselli G., Zangrillo A., Zanella A., Antonelli M., Cabrini L., Castelli A., Cereda D., Coluccello A., Foti G., Fumagalli R. (2020). Baseline Characteristics and Outcomes of 1591 Patients Infected With SARS-CoV-2 Admitted to ICUs of the Lombardy Region, Italy. JAMA.

[7] Sinha P., Calfee C.S., Cherian S., Brealey D., Cutler S., King C., Killick C., Richards O., Cheema Y., Bailey C. (2020). Prevalence of phenotypes of acute respiratory distress syndrome in critically ill patients with COVID-19: A prospective observational study. Lancet Respir. Med..

[8] Gattinoni L., Coppola S., Cressoni M., Busana M., Rossi S., Chiumello D. (2020). COVID-19 Does Not Lead to a “Typical” Acute Respiratory Distress Syndrome. Am. J. Respir. Crit. Care Med..

[9] Grasselli G., Tonetti T., Protti A., Langer T., Girardis M., Bellani G., Laffey J., Carrafiello G., Carsana L., Rizzuto C. (2020). Pathophysiology of COVID-19-associated acute respiratory distress syndrome: A multicentre prospective observational study. Lancet Respir. Med..

[10] George P.M., Wells A.U., Jenkins R.G. (2020). Pulmonary fibrosis and COVID-19: The potential role for antifibrotic therapy. Lancet Respir. Med..

[11] Marchioni A., Tonelli R., Rossi G., Spagnolo P., Luppi F., Cerri S., Cocconcelli E., Pellegrino M.R., Fantini R., Tabbìet L. (2020). Ventilatory support and mechanical properties of the fibrotic lung acting as a “squishy ball”. Ann. Intensive Care.

[12] Tay M.Z., Poh C.M., Rénia L., MacAry P.A., Ng L.F.P. (2020). The trinity of COVID-19: Immunity, inflammation and intervention. Nat. Rev. Immunol..

[13] Laing A.G., Lorenc A., Barrio I.d.M.d., Das A., Fish M., Monin L., Muñoz-Ruiz M., McKenzie D.R., Hayday T.S., Francos-Quijorna I.A. (2020). dynamic COVID-19 immune signature includes associations with poor prognosis. Nat. Med..

[14] Mehta P., McAuley D.F., Brown M., Sanchez E., Tattersall R.S., Manson J.J. (2020). COVID-19: Consider cytokine storm syndromes and immunosuppression. Lancet.

[15] Iba T., Levy J.H., Levi M., Connors J.M., Thachil J. (2020). Coagulopathy of Coronavirus Disease 2019. Crit. Care Med..

[16] Varga Z., Flammer A.J., Steiger P., Haberecker M., Andermatt R., Zinkernagel A.S., Mehra M.R., Schuepbach R.A., Ruschitzka F., Moch H. (2020). Endothelial cell infection and endotheliitis in COVID-19. Lancet.

[17] Nimmerjahn F., Ravetch J.V. (2008). Fcγ receptors as regulators of immune responses. Nat. Rev. Immunol..

[18] Peiris J.S.M., Chu C.M., Cheng V.C.C., Chan K.S., Hung I.F.N., Poon L.L.M., Law K.I., Tang B.S.F., Hon T.Y.W., Chan C.S. (2003). Clinical progression and viral load in a community outbreak of coronavirus-associated SARS pneumonia: A prospective study. Lancet.

[19] Yoshida T., Uchiyama A., Matsuura N., Mashimo T., Fujino Y. (2013). The Comparison of Spontaneous Breathing and Muscle Paralysis in Two Different Severities of Experimental Lung Injury*. Crit. Care Med..

[20] Fernandez I.E., Eickelberg O. (2012). New cellular and molecular mechanisms of lung injury and fibrosis in idiopathic pulmonary fibrosis. Lancet.

[21] Burnham E.L., Janssen W.J., Riches D.W.H., Moss M., Downey G.P. (2014). The fibroproliferative response in acute respiratory distress syndrome: Mechanisms and clinical significance. Eur. Respir. J..

[22] Sgalla G., Cocconcelli E., Tonelli R., Richeldi L. (2016). Novel drug targets for idiopathic pulmonary fibrosis. Expert Rev. Respir. Med..

[23] Hamada N., Kuwano K., Yamada M., Hagimoto N., Hiasa K., Egashira K., Nakashima N., Maeyama T., Yoshimi M., Nakanishi Y. (2005). Anti-Vascular Endothelial Growth Factor Gene Therapy Attenuates Lung Injury and Fibrosis in Mice. J. Immunol..

[24] Wang Y., Maciejewski B.S., Soto-Reyes D., Lee H.-S., Warburton D., Sanchez-Esteban J. (2009). Mechanical stretch promotes fetal type II epithelial cell differentiation via shedding of HB-EGF and TGF-α. J. Physiol..

[25] Cabrera-Benítez N.E., Parotto M., Post M., Han B., Spieth P.M., Cheng W.E., Valladares F., Villar J., Liu M., Sato M. (2012). Mechanical stress induces lung fibrosis by epithelial–mesenchymal transition*. Crit. Care Med..

[26] Putensen C., Zech S., Wrigge H., Zinserling J., Stuber F., VON SPIEGEL T.I.L.M.A.N.N., Mutz N. (2001). Long-term effects of spontaneous breathing during ventilatory support in patients with acute lung injury. Am. J. Respir. Crit. Care Med..

[27] Goligher E.C., Dres M., Patel B.K., Sahetya S.K., Beitler J.R., Telias I., Yoshida T., Vaporidi K., Grieco D.L., Schepens T. (2020). Lung- and Diaphragm-Protective Ventilation. Am. J. Respir. Crit. Care Med..

[28] Mascheroni D., Kolobow T., Fumagalli R., Moretti M.P., Chen V., Buckhold D. (1988). Acute respiratory failure following pharmacologically induced hyperventilation: An experimental animal study. Intensive Care Med..

[29] Tonelli R., Fantini R., Tabbì L., Castaniere I., Pisani L., Pellegrino M.R., Casa G.D., D’Amico R., Girardis M., Nava S. (2020). Early Inspiratory Effort Assessment by Esophageal Manometry Predicts Noninvasive Ventilation Outcome in De Novo Respiratory Failure. A Pilot Study. Am. J. Respir. Crit. Care Med..

[30] Brochard L., Slutsky A., Pesenti A. (2016). Mechanical Ventilation to Minimize Progression of Lung Injury in Acute Respiratory Failure. Am. J. Respir. Crit. Care Med..

[31] Goligher E.C., Fan E., Herridge M.S., Murray A., Vorona S., Brace D., Rittayamai N., Lanys A., Tomlinson G., Singh J.M. (2015). Evolution of Diaphragm Thickness during Mechanical Ventilation. Impact of Inspiratory Effort. Am. J. Respir. Crit. Care Med..

[32] Spinelli E., Mauri T., Beitler J.R., Pesenti A., Brodie D. (2020). Respiratory drive in the acute respiratory distress syndrome: Pathophysiology, monitoring, and therapeutic interventions. Intensive Care Med..

[33] Jacono F.J., Peng Y.J., Nethery D., Faress J.A., Lee Z., Kern J.A., Prabhakar N.R. (2006). Acute lung injury augments hypoxic ventilatory response in the absence of systemic hypoxemia. J. Appl Physiol..

[34] Lin S., Walker J., Xu L., Gozal D., Yu J. (2007). Behaviours of pulmonary sensory receptors during development of acute lung injury in the rabbit. Exp. Physiol..

[35] Meinhardt J., Radke J., Dittmayer C., Franz J., Thomas C., Mothes R., Laue M., Schneider J., Brünink S., Greuel S. (2021). Olfactory transmucosal SARS-CoV-2 invasion as a port of central nervous system entry in individuals with COVID-19. Nat. Neurosci..

[36] Yoshida T., Brochard L. (2018). Esophageal pressure monitoring: Why, when and how?. Curr. Opin. Crit. Care.

[37] D’Angelo E., Agostoni E. (1973). Continuous recording of pleural surface pressure at various sites. Respir. Physiol..

[38] Minh V.D., Friedman P.J., Kurihara N., Moser K.M. (1974). Ipsilateral transpulmonary pressures during unilateral electrophrenic respiration. J. Appl. Physiol..

[39] Bhattacharya M., Kallet R.H., Ware L.B., Matthay M.A. (2016). Negative-Pressure Pulmonary Edema. Chest.

[40] Yoshida T., Nakahashi S., Nakamura MA M., Koyama Y., Roldan R., Torsani V., De Santis R.R., Gomes S., Uchiyama A., Amato M.B.P. (2017). Volume-controlled Ventilation Does Not Prevent Injurious Inflation during Spontaneous Effort. Am. J. Respir. Crit. Care Med..

[41] Xie J., Tong Z., Guan X., Du B., Qiu H., Slutsky A.S. (2020). Critical care crisis and some recommendations during the COVID-19 epidemic in China. Intensive Care Med..

[42] Guan W.J., Ni Z.Y., Hu Y., Liang W.H., Ou C.Q., He J.X., Liu L., Shan H., Lei C.L., Hui D.S. (2020). Clinical Characteristics of Coronavirus Disease 2019 in China. N. Engl. J. Med..

[43] Recasens B.B., Martinez-Llorens J.M., Rodriguez-Sevilla J.J., Rubio M.A. (2020). Lack of dyspnea in patients with Covid-19: Another neurological conundrum?. Eur. J. Neurol..

[44] Esnault P., Cardinale M., Hraiech S., Goutorbe P., Baumstrack K., Prud’homme E., Bordes J., Forel J.-M., Meaudre E., Papazian L. (2020). High respiratory drive and excessive respiratory efforts predict relapse of respiratory failure in critically ill patients with COVID-19. Am. J. Respir. Crit. Care Med..

[45] Nahama A., Ramachandran R., Cisternas A.F., Ji H. (2020). The role of afferent pulmonary innervation in ARDS associated with COVID-19 and potential use of resiniferatoxin to improve prognosis: A review. Med Drug Discov..

[46] Tipton M.J., Harper A., Paton J.F.R., Costello J.T. (2017). The human ventilatory response to stress: Rate or depth?. J. Physiol..

[47] Grieco D.L., Menga L.S., Eleuteri D., Antonelli M. (2019). Patient self-inflicted lung injury: Implications for acute hypoxemic respiratory failure and ARDS patients on non-invasive support. Minerva Anestesiol..

[48] Tonelli R., Tabbì L., Fantini R., Castaniere I., Gozzi F., Busani S., Nava S., Clini E., Marchioni A. (2020). Reply to Tuffet et al. and to Michard and Shelley. Am. J. Respir. Crit. Care Med..

[49] Grieco D.L., Menga L.S., Raggi V., Bongiovanni F., Anzellotti G.M., Tanzarella E.S., Bocci M.G., Mercurio G., Dell’Anna A.M., Eleuteri D. (2020). Physiological Comparison of High-Flow Nasal Cannula and Helmet Noninvasive Ventilation in Acute Hypoxemic Respiratory Failure. Am. J. Respir. Crit. Care Med..

[50] L’Her E., Deye N., Lellouche F., Taille S., Demoule A., Fraticelli A., Mancebo J., Brochard L. (2005). Physiologic Effects of Noninvasive Ventilation during Acute Lung Injury. Am. J. Respir Crit. Care Med..

[51] National Heart, Lung, and Blood Institute PETAL Clinical Trials Network (2019). Early Neuromuscular Blockade in the Acute Respiratory Distress Syndrome. N. Engl. J. Med..

[52] Morais C.C., Koyama Y., Yoshida T., Plens G.M., Gomes S., Lima C.A., Ramos O.P.S., Pereira S.M., Kawaguchi N., Yamamoto H. (2018). High Positive End-Expiratory Pressure Renders Spontaneous Effort Noninjurious. Am. J. Respir. Crit. Care Med..

[53] Kiss T., Bluth T., Braune A., Huhle R., Denz A., Herzog M., Herold J., Vivona L., Millone M., Bergamaschi A. (2019). Effects of Positive End-Expiratory Pressure and Spontaneous Breathing Activity on Regional Lung Inflammation in Experimental Acute Respiratory Distress Syndrome. Crit. Care Med..

[54] Yoshida T., Grieco D.L., Brochard L., Fujino Y. (2020). Patient self-inflicted lung injury and positive end-expiratory pressure for safe spontaneous breathing. Curr. Opin. Crit. Care.

[55] Marchioni A., Tonelli R., Fantini R., Tabbì L., Castaniere I., Livrieri F., Bedogni S., Ruggieri V., Pisani L., Nava S. (2019). Respiratory Mechanics and Diaphragmatic Dysfunction in COPD Patients Who Failed Non-Invasive Mechanical Ventilation. Int. J. Chron. Obs. Pulmon. Dis..

[56] Laghi F., Harrison M.J., Tobin M.J. (1996). Comparison of magnetic and electrical phrenic nerve stimulation in assessment of diaphragmatic contractility. J. Appl. Physiol..

[57] De Troyer A., Leduc D., Cappello M., Mine B., Gevenois P.A., Wilson T.A. (2009). Mechanisms of the inspiratory action of the diaphragm during isolated contraction. J. Appl. Physiol..

[58] Tonelli R., Castaniere I., Fantini R., Tabbì L., Busani S., Pisani L., Nava S., Clini E., Marchioni A. (2020). Reply to Spinelli et al. and to Jha: Continued Vigorous Inspiratory Effort as a Predictor of Noninvasive Ventilation Failure. Am. J. Respir. Crit. Care Med..

[59] Marchioni A., Tonelli R., Ball L., Fantini R., Castaniere I., Cerri S., Luppi F., Malerba M., Pelosi P., Clini E. (2018). Acute exacerbation of idiopathic pulmonary fibrosis: Lessons learned from acute respiratory distress syndrome?. Crit. Care.

[60] Fernández-Pérez E.R., Yilmaz M., Jenad H., Daniels C.E., Ryu J.H., Hubmayr R.D., Gajic O. (2008). Ventilator settings and outcome of respiratory failure in chronic interstitial lung disease. Chest.

[61] Thompson A.E., Ranard B.L., Wei Y., Jelic S. (2020). Prone Positioning in Awake, Nonintubated Patients With COVID-19 Hypoxemic Respiratory Failure. JAMA Intern. Med..

[62] Rochwerg B., Brochard L., Elliott M.W., Hess D., Hill N.S., Nava S., Navalesi P., Antonelli M., Brozek J., Conti G. (2017). Official ERS/ATS clinical practice guidelines: Noninvasive ventilation for acute respiratory failure. Eur. Respir. J..

[63] Alraddadi B.M., Qushmaq I., Al-Hameed F.M., Mandourah Y., Almekhlafi G.A., Jose J., Al-Omari A., Kharaba A., Almotairi A., Khatib K.A. (2019). Noninvasive ventilation in critically ill patients with the Middle East respiratory syndrome. Influenza Other Respir. Viruses.

[64] Cheung T.M., Yam L.Y., So L.K., Lau A.C., Poon E., Kong B.M., Yung R.W. (2004). Effectiveness of Noninvasive Positive Pressure Ventilation in the Treatment of Acute Respiratory Failure in Severe Acute Respiratory Syndrome. Chest.

[65] Wang D., Hu B., Hu C., Zhu F., Liu X., Zhang J., Wang B., Xiang H., Cheng Z., Xiong Y. (2020). Clinical Characteristics of 138 Hospitalized Patients With 2019 Novel Coronavirus–Infected Pneumonia in Wuhan, China. JAMA.

[66] Yang X., Yu Y., Xu J., Shu H., Liu H., Wu Y., Zhang L., Yu Z., Fang M., Yu T. (2020). Clinical course and outcomes of critically ill patients with SARS-CoV-2 pneumonia in Wuhan, China: A single-centered, retrospective, observational study. Lancet Respir. Med..

[67] Franco C., Facciolongo N., Tonelli R., Dongilli R., Vianello A., Pisani L., Scala R., Malerba M., Carlucci A., Negri E.A. (2020). Feasibility and clinical impact of out-of-ICU non-invasive respiratory support in patients with COVID-19 related pneumonia. Eur. Respir. J..

[68] Demoule A., Vieillard Baron A., Darmon M., Beurton A., Géri G., Voiriot G., Dupont T., Zafrani L., Girodias L., Labbé V. (2020). High-Flow Nasal Cannula in Critically III Patients with Severe COVID-19. Am. J. Respir. Crit. Care Med..

[69] Protti A., Andreis D.T., Monti M., Santini A., Sparacino C.C., Langer T., Votta E., Gatti S., Lombardi L., Leopardi O. (2013). Lung stress and strain during mechanical ventilation: Any difference between statics and dynamics?. Crit. Care Med..

[70] Horio Y., Takihara T., Niimi K., Komatsu M., Sato M., Tanaka J., Takiguchi H., Tomomatsu H., Tomomatsu K., Hayama N. (2016). High-flow nasal cannula oxygen therapy for acute exacerbation of interstitial pneumonia: A case series. Respir. Investig..

[71] Arentz M., Yim E., Klaff L., Lokhandwala S., Riedo F.X., Chong M., Lee M. (2020). Characteristics and Outcomes of 21 Critically Ill Patients With COVID-19 in Washington State. JAMA.

[72] Huang C., Wang Y., Li X., Ren L., Zhao J., Hu Y., Zhang L., Fan G., Xu J., Gu X. (2020). Clinical features of patients infected with 2019 novel coronavirus in Wuhan, China. Lancet.

[73] Wang K., Zhao W., Li J., Shu W., Duan J. (2020). The experience of high-flow nasal cannula in hospitalized patients with 2019 novel coronavirus-infected pneumonia in two hospitals of Chongqing, China. Ann. Intensive Care.

[74] Chen N., Zhou M., Dong X., Qu J., Gong F., Han Y., Qiu Y., Wang J., Liu Y., Wei Y. (2020). Epidemiological and clinical characteristics of 99 cases of 2019 novel coronavirus pneumonia in Wuhan, China: A descriptive study. Lancet.

[75] Zhou F., Yu T., Du R., Fan G., Liu Y., Liu Z., Xiang J., Wang Y., Song B., Gu X. (2020). Clinical course and risk factors for mortality of adult inpatients with COVID-19 in Wuhan, China: A retrospective cohort study. Lancet.

[76] Wu C., Chen X., Cai Y., Xia J., Zhou X., Xu S., Huang H., Zhang L., Zhou X., Du C. (2020). Risk Factors Associated with Acute Respiratory Distress Syndrome and Death in Patients With Coronavirus Disease 2019 Pneumonia in Wuhan, China. JAMA Intern Med..

[77] Bhatraju P.K., Ghassemieh B.J., Nichols M., Kim R., Jerome K.R., Nalla A.K., Greninger A.L., Pipavath S., Wurfel M.M., Evans L. (2020). Covid-19 in Critically Ill Patients in the Seattle Region—Case Series. N. Engl. J. Med..

[78] Aliberti S., Radovanovic D., Billi F., Sotgiu G., Costanzo M., Pilocane T., Saderi L., Gramegna A., Rovellini A., Perotto L. (2020). Helmet CPAP treatment in patients with COVID-19 pneumonia: A multicenter, cohort study. Eur. Respir. J..

[79] Alhazzani W., Møller M.H., Arabi Y.M., Loeb M., Gong M.N., Fan E., Oczkowski S., Levy M.M., Derde L., Dzierba A. (2020). Surviving Sepsis Campaign: Guidelines on the Management of Critically Ill Adults with Coronavirus Disease 2019 (COVID-19). Crit. Care Med..

[80] Cornejo R.A., Díaz J.C., Tobar E.A., Bruhn A.R., Ramos C.A., González R.A., Repetto C.A., Romero C.M., Gálvez L.R., Llanos O. (2013). Effects of Prone Positioning on Lung Protection in Patients with Acute Respiratory Distress Syndrome. Am. J. Respir. Crit. Care Med..

[81] Nyrén S., Radell P., Lindahl S.G.E., Mure M., Petersson J., Larsson S.A., Jacobsson H., Sánchez-Crespo A. (2010). Lung ventilation and perfusion in prone and supine postures with reference to anesthetized and mechanically ventilated healthy volunteers. Anesthesiology.

[82] Raoof S., Nava S., Carpati C., Hill N.S. High-Flow, Noninvasive Ventilation and Awake (Nonintubation) Proning in Patients With COVID-2019 With Respiratory Failure. Chest.

[83] Coppo A., Bellani G., Winterton D., Di Pierro M., Soria A., Faverio P., Cairo M., Mori S., Messinesi G., Contro E. (2020). Feasibility and physiological effects of prone positioning in non-intubated patients with acute respiratory failure due to COVID-19 (PRON-COVID): A prospective cohort study. Lancet Respir. Med..

[84] Nakos G., Tsangaris I., Kostanti E., NATHANAIL C., Lachana A., Koulouras V., Kastani D. (2000). Effect of the Prone Position on Patients with Hydrostatic Pulmonary Edema Compared with Patients with Acute Respiratory Distress Syndrome and Pulmonary Fibrosis. Am. J. Respir. Crit. Care Med..

